# *Hesperentomon yangi* sp. n. from Jiangsu Province, Eastern China, with analyses of DNA barcodes (Protura, Acerentomata, Hesperentomidae)

**DOI:** 10.3897/zookeys.338.6099

**Published:** 2013-10-02

**Authors:** Yi Bai, Yun Bu

**Affiliations:** 1 School of Life Sciences, Taizhou University, Taizhou, 317000, China; 2 Institute of Zoology, Shaanxi Normal University, Xi’an, 710062, China; 3 Institute of Plant Physiology & Ecology, Shanghai Institutes for Biological Sciences, Chinese Academy of Sciences, Shanghai 200032, China

**Keywords:** Protura, *Hesperentomon*, molecular data, genetic divergence, chaetotaxy

## Abstract

*Hesperentomon yangi* **sp. n.** is described from eastern China. Its DNA barcodes are sequenced and compared to the similar species of the genus. *Hesperentomon yangi* **sp. n.** is characterized by 12 posterior setae on tergites II–VI, 8 posterior setae on sternites IV–VI (seta *Pc* absent), absence of seta *sd4* on head, absence of seta *P2a* on tergite VII, 6 and 8 anterior setae on mesosternum and metasternum respectively, and few teeth on comb. It differs from *Hesperentomon xiningense* Bu & Yin, 2007 and *Hesperentomon nanshanensis* Bu & Yin, 2007 in the chaetotaxy of mesosternum and metanotum, maxillary gland, length and shape of some sensilla on foretarsus, as well as the body porotaxy. The genetic divergences of DNA barcodes sequences between *Hesperentomon yangi* **sp. n.**, *Hesperentomon xiningense* and *Hesperentomon nanshanensis* are 24.1% on average, which is distinctly higher than the divergences between individuals of the new species (0.5%). Molecular data provide a solid evidence of the new species identified by the morphological characters.

## Introduction

The genus *Hesperentomon* Price, 1960 currently contains 17 species, which with 14 species have been found in China (Bu and Yin 2007a, 2007b, Bu et al. 2011, Shrubovych 2010, Szeptycki 2007, Wu and Yin 2008, Yin 1999). During a collection from Qixia Mountain, Nanjing City, East China (Jiangsu Province), some specimens of the genus *Hesperentomon* were first found from that area. They were identified as a new species and described as *Hesperentomon yangi* sp. n. in the present paper. In order to confirm the morphological identification, the DNA barcodes of the new species and two similar congeners *Hesperentomon xiningense* and *Hesperentomon nanshanensis* were sequenced and analyzed.

## Materials and methods

The specimens were collected with Tullgren funnels. All specimens were mounted on slides in Hoyer’s medium and dried for three days in an oven at 60°C. Specimens were identified and drawn with the aid of a NIKON E600 phase contrast microscope. Type specimens are deposited in the Shanghai Entomological Museum (SEM), Institute of Plant Physiology & Ecology, Shanghai Institutes for Biological Sciences, Chinese Academy of Sciences.

Abbreviations used in the text follow the paper of Bu and Yin (2007b). Head setae and pores are marked according to Rusek et al. (2012).

For DNA barcodes, genomic DNA was extracted from each individual separately by means of a non-destructive method (after Gilbert et al. 2007) with minor modifications. The information for the species is given in Table 1. After the DNA extraction, the cuticles of proturans were retrieved and mounted on the slides as voucher specimens. DNA barcoding sequences of mitochondrial COI gene were amplified and sequenced by primer pair LCO/HCO (Folmer et al. 1994). The barcodes sequences are deposited in GenBank. The genetic divergence and nucleotide composition were calculated using MEGA version 5 (Tamura et al. 2011).

**Table 1.** Information for the species used in the study.

**Classification**	**Species**	**Locality**	**Number of individuals**	**GenBank Accession Numbers**
**Protura**				
**Acerentomata**				
**Hesperentomidae**				
***Hesperentomon***	*Hesperentomon yangi* sp. n.	China: Jiangsu	3	KF530824, KF530825, KF530826
	*Hesperentomon xiningense*	China: Qinghai	1	KF530827
	*Hesperentomon nanshanensis*	China: Qinghai	1	KF530828

## Results

### Taxonomy

#### Hesperentomon yangi sp. n.

http://zoobank.org/B4190939-D197-4E2E-ACF3-2A7635941DC3

http://species-id.net/wiki/Hesperentomon_yangi

Figs 1 – 25 , Table 2

##### Material examined.

Holotype, female (No. NJ-8), East China, Jiangsu Province, Nanjing City, extracted from the soil samples under some big trees of Qixia Mountain, 32°09.45'N, 118°57.60'E, elev. 200 m, 29-XI-2012. coll. Y. M. Yang. Paratype, 6 females (Nos. NJ-2, NJ-3, NJ-4, NJ-7, NJ-12, NJ-13), 6 males (Nos. NJ-1, NJ-5, NJ-6, NJ-9, NJ-10, NJ-11), same data as holotype. Other materials, 1 maturus junior (No. NJ-14) and 1 larva II (No. NJ-15). Specimens NJ-13, NJ-14 and NJ-15 are voucher specimens with DNA barcodes sequenced. Type specimens are deposited in Shanghai Entomological Museum (SEM), Institute of Plant Physiology & Ecology, Chinese Academy of Sciences.

##### Description.

**Figures 1–11.**
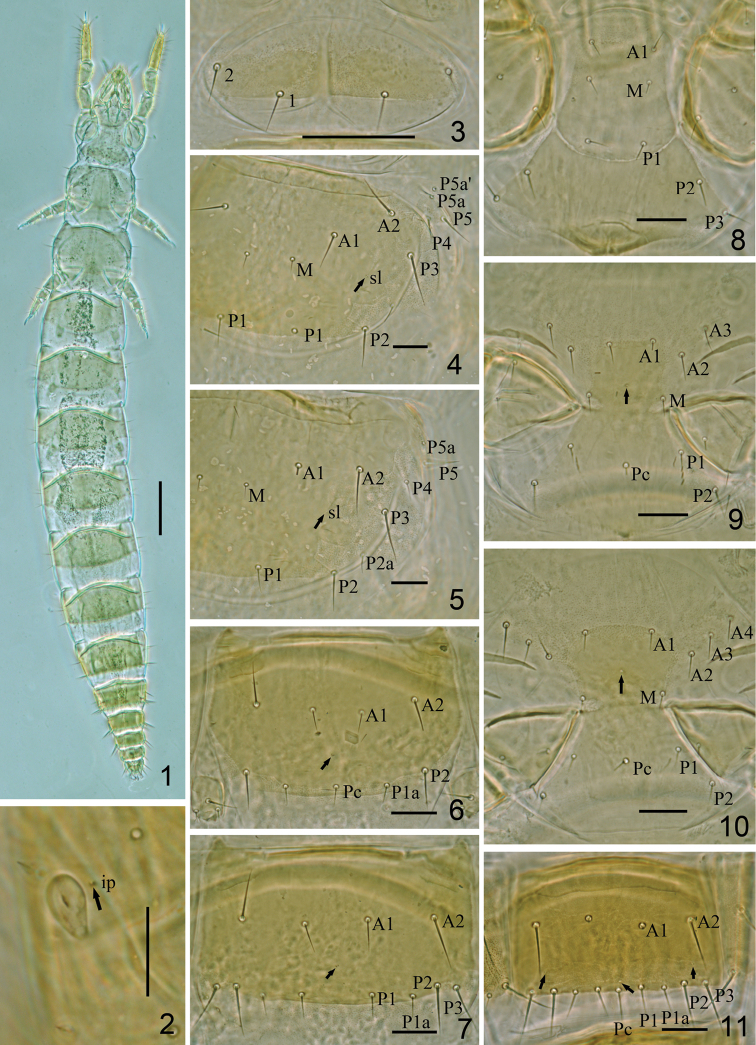
*Hesperentomon yangi* sp. n. Holotype. **1** Habitus **2** pseudoculus **3** pronotum **4** mesonotum (*sl*=sublateral pore) **5** metanotum **6** sternite II **7** sternite IV **8** prosternum **9** mesosternum **10** metasternum **11** sternite VII. Arrows show pores. Scale bar: 100 μm in Fig. **1**, others, 20 μm.

Adult body length 1300–1400 µm (n=13), yellow-brown, and foretarsus with deeper color (Fig. 1).

**Figures 12–22.**
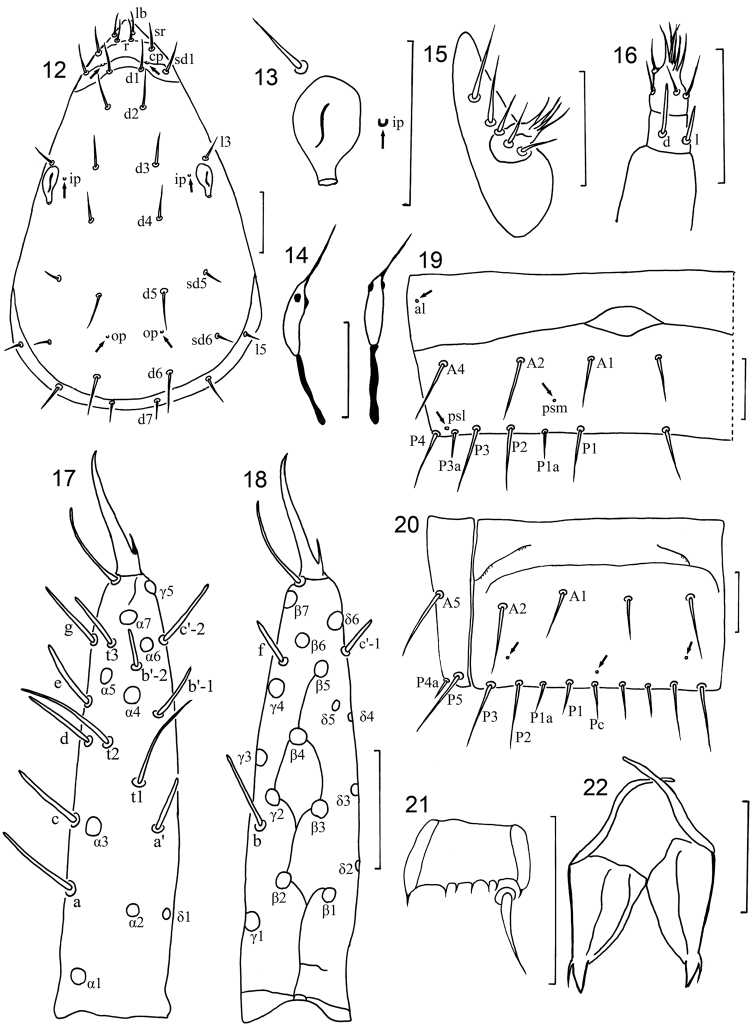
*Hesperentomon yangi* sp. n. Holotype. **12** Head, dorsal view (*cp*=clypeal pores, *ip*=interpseudocular pores, *op* =occipital pores) **13** pseudoculus **14** canal of maxillary gland **15** labial palpus **16** maxillary palpus, **17** foretarsus, exterior view **18** foretarsus, interior view **19** tergite VII, left side (*al*=anterolateral pore, *psm*=posterosubmedial pore, *psl*=posterosublateral pore) **20** sternite VII and lateral part of tergite VII **21** Comb **22** female squama genitalis. Arrows show pores. Scale bar: 20 μm.

**Head.** Oblong, length 125–130 µm, width 85–95 µm. Dorsal setae long, other setae short. Setae *d6* and *sd6* present, *sd4* absent. Seta *d6* 14–15 µm, *d7* 6–7 µm in length. Paired pores *cp*, *ip* and *op* present, pore *fp* absent (Fig. 12). Pseudoculus pear-shaped, with short posterior extension, length 12–15 µm, width 8–9 µm. PR=9–11 (Figs 2, 13). Canal of maxillary gland with sausage-like calyx, posterior dilation about equal to length of calyx. CF=7–9 (Fig. 14). Labial palpus well developed, without basal sensillum (Fig. 15). Maxillary palpus with two tapering sensilla, dorsal one (8–9 µm) distinctly longer than lateral one (5–6 µm) (Fig. 16).

**Foretarsus**. Length 73–83 µm, claw length 18–23 µm, without inner flap, TR=3.5–4.4; empodium length 3–4 µm, EU=0.14–0.18. Dorsal sensilla *t-1* and *t-2* slender and long (14–15 µm), BS=1.1; *t-3* slengder, not reaching base of claw. Exterior sensilla all sward-like and in different length, *a* surpassing base of *c*, *b* and *c* subequal in length, *d* close to *t2*, *e* broad, *f* short, *g* long. Interior sensilla also sward-like in different length, *a*’ short, *b’-1* longer than *b’-2*, *c’-2* longer than *c’-1*. Relative length of sensilla: *b’-2* < *c’-1* < *f*< *a*’< *t3*< *b’-1*< (*b*=*c*=*e*) < *d*<(*g*= *c’-2*) < *a* < *t2* < *t1* (Figs 17, 18). Length of middle tarsus 35–37 µm, claw length 18–20 µm. Length of hind tarsus 38–40 µm, claw length 20–23 µm.

**Thorax.** Thoracic chaetotaxy given in Table 2. Setae *1* and *2* on pronotum subequal in length; mesonotum with eight pairs of posterior setae, *P5a* and *P5a*’ minute; metanotum with seven pairs of posterior setae, *P5a* minute; setae *P1* and *P2* on mesonotum 13–15 µm and 17–20 µm respectively. (Figs 3–5). Prosternum without anterior seta *A2*, mesosternum and metasternum with 6 and 8 anterior setae respectively (Figs 8–10). All setae on thoracic sternites setiform. Pronotum and prosternum without pores. Mesonotum and metanotum with pores *sl* (Figs 4, 5). Mesosternum and metasternum each with single median pore, situated anterior to level of setae *M* (Figs 9, 10).

**Table 2.** Adult chaetotaxy of *Hesperentomon yangi* sp. n.

**Segment**		**Dorsal**		**Ventral**	
		**Formula**	**Setae**	**Formula**	**Setae**
Thorax	I	4	1, 2	(2-2)/6	A1, M, P1, 2, 3
	II	6/4	A2, 4, M, P1, 2, 2a, 3, 4, 5, 5a, 5a’	(6-2)/5	A1, 2, 3, M, Pc, 1, 2
	III	6/14	A2, 4, M, P1, 2, 2a, 3, 4, 5, 5a	(8-2)/5	A1, 2, 3, 4, M, Pc, 1, 2
Abdomen	I	4/10	A1, 2, P1, 2, 3, 4, 5	4/4	A1, 2, P1, 2
	II–III	8/12	A1, 2, 4, 5, P1, 2, 3, 4, 4a, 5	4/5	A1, 2, Pc, 1a, 2
	IV–VI	8/12	A1, 2, 4, 5, P1, 2, 3, 4, 4a, 5	4/8	A1, 2, P1, 1a, 2, 3
	VII	8/16	A1, 2, 4, 5, P1, 1a, 2, 3, 3a, 4, 4a, 5	4/9	A1, 2, Pc, 1, 1a, 2, 3
	VIII	6/14	A1, 2, 5, P1, 1a, 2, 2a, 3, 3a, 5	6	1, 1a, 2
	IX	12	1, 2, 2a, 3, 3a, 4	6	1, 1a, 2
	X	10	1, 2, 3, 3a, 4	6	1, 1a, 2
	XI	8	1, 2, 3, 4	6	1, 1a, 2
	XII	9		8	

**Figures 23–25.**
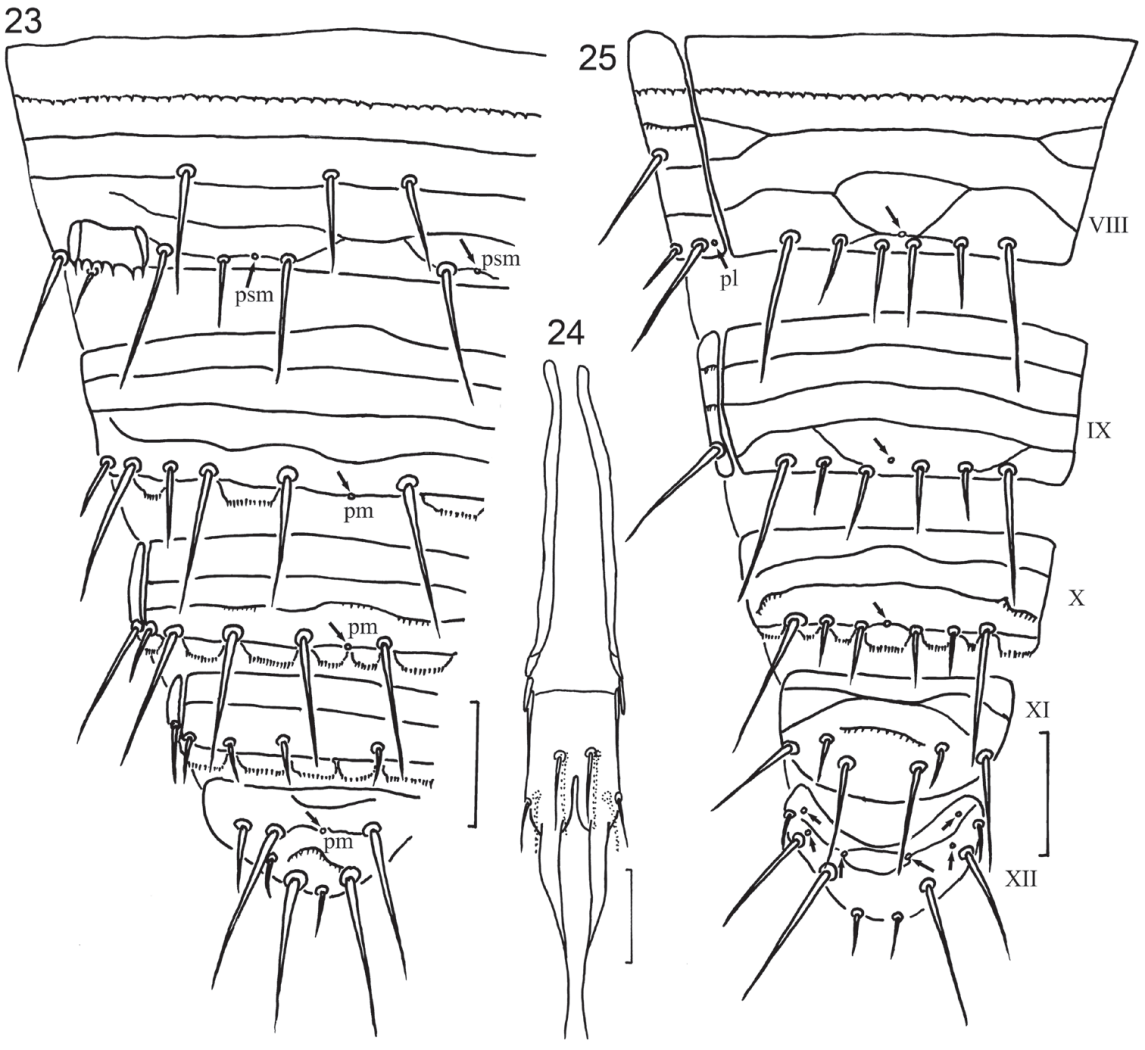
*Hesperentomon yangi* sp. n. **23** Tergites VIII–XII, left side (*pm*= posteromedial pore) **24** male squama genitalis **25** sternites VIII–XII (*pl*=posterolateral pore) **23** and **25** holotype **24** paratype NJ-10. Arrows show pores. Scale bar: 20 μm.

**Abdomen.** Abdominal chaetotaxy given in Table 2. Tergite I with two pairs of anterior setae (*A1*, *A2*) and five pairs of posterior setae. Tergites II–VI with four pairs of anterior setae (*A1*, *A2*, *A4*, *A5*) and six pairs of posterior setae, *P1a*, *P2a* and *P3a* absent. Tergite VII with 8 posterior setae, *P2a* absent. Posterior central seta *Pc* absent on sternites IV–VI (8 posterior setae) (Fig. 7), present on sternite VII (Figs 11, 20). Bases of setae on segments X–XII without surrounding ciliation. Tergites IX–XI, and sternite X with broad, coarsely serrated posterior lamella (Figs 23, 25).

Tergites I–VI with pores *psm* and *al*, VII with pores *psm*, *psl* and *al* (Fig. 19), VIII with pores *psm* and *pl*, IX, X and XII each with single posteromedial pore (*pm*), XI without pores (Figs 23, 25). Sternites I–VI each with one medial pore (Fig. 6), VII with one medial pore and one pair of lateral pores (Figs 11, 20), VIII–X each with single medial pore, XI without pores, XII with 3+3 pores.

Abdominal appendages typical of the genus, each with two segments and four setae. Striate band on abdominal segment VIII reduced and only single serrate line present (Fig. 23). Comb on abdomen VIII rectangular, with 7–8 teeth (Fig. 21).

Female squama genitalis robust. Each acrostylus with one slender flap on its outer side (Fig. 22). Male squama genitalis with 2+2 setae on dorsal side and 2+2 setae on ventral side (Fig. 24).

##### Etymology.

The species is named after Mr. Yi-Ming Yang who collected the specimens and in remembrance of his great contribution to the collection of Protura in China.

##### Chaetal variability.

Chaetal variations were observed in 5 specimens: on pronotum, with 5 seate, presence of 1 additional seta on left side (No. NJ-2); on prosternum, asymmetrical absence of *A1* of right side (No. NJ-3); on sternite I, presence of *Pc* (Nos. NJ-4, NJ-6, NJ-12); on sternite IV and V, presence of *Pc* (No. NJ-12).

##### Distribution.

Jiangsu (Nanjing), China.

##### Diagnosis.

*Hesperentomon yangi* sp. n. is characterized by 12 posterior setae on tergites II–VI, 8 posterior setae on sternites IV–VI (seta *Pc* absent), absence of seta *sd4* on head, absence of seta *P2a* on tergite VII, 6 and 8 anterior setae on mesosternum and metasternum respectively, and few teeth on comb.

##### Remarks.

*Hesperentomon yangi* sp. n. is similar to *Hesperentomon xiningense* Bu & Yin, 2007 and *Hesperentomon nanshanensis* Bu & Yin, 2007 in having 8 posterior setae on sternites IV–VI (seta *Pc* absent), 12 posterior setae on tergite IV–VI, and the absence of seta *P2a* on tergite VII. It can be distinguished from those two species by the chaetotaxy of mesosternum and metasternum (6 and 8 anterior setae in *Hesperentomon yangi* sp. n. respectively vs. 4 and 6 anterior setae in *Hesperentomon xiningense* and *Hesperentomon nanshanensis*), chaetotaxy of head (setae *sd4* absent in *Hesperentomon yangi* sp. n. vs. present in the later two species), porotaxy of head (frontal pores *fp* absent and interpseudocular pores *ip* present in *Hesperentomon yangi* sp. n. vs. *fp* present and *ip* absent in the later two), porotaxy of sternite VII (3 pores in *Hesperentomon yangi* sp. n. vs. 1 pore in the later two). It also differs from *Hesperentomon xiningense* in the length of foretarsal sensillum *b* subequal length to *c* (*b* distinctly longer than *c* in *Hesperentomon xiningense*), short sensillum *b’-2* which not reaching base of seta *α7* (*b’-2* surpassing base of seta *α7* in *Hesperentomon xiningense*), and the presence of regular teeth on hind margin of striate band (with sparse unregular teeth in *Hesperentomon xiningense*). It also differs from *Hesperentomon nanshanensis* in the chaetotaxy of abdominal segment X (10 and 6 setae on tergite and sternite repectively in *Hesperentomon yangi* sp. n. vs. 8 and 4 setae in *Hesperentomon nanshanensis*) and shape of maxillary gland (posterior dilation about equal length of the calyx in *Hesperentomon yangi* sp. n. vs. about 1/2 length of the calyx in *Hesperentomon nanshanensis*).

### The DNA barcodes analyses

The standard DNA barcoding sequence (COI gene) from 3 individuals (Nos. NJ-13, NJ-14, and NJ-15) of *Hesperentomon yangi* sp. n., 1 individual of *Hesperentomon xiningense* and 1 individual of *Hesperentomon nanshanensis* were sequenced and deposited in GenBank. The accession numbers given in Table 1. Except a 6 base pairs deletion was found in *Hesperentomon xiningense* (652 base pairs), other sequences each contains 658 base pairs. The nucleotide compositions as A=34.3%, T=33.6%, C=18.9%, G=13.1% on average in *Hesperentomon yangi* sp. n., A=27.0%, T=35.5%, C=22.4%, G=15.1% in *Hesperentomon xiningense*, and A=29.7%, T=35.8%, C=20.2%, G=14.3% in *Hesperentomon nanshanensis*. The genetic divergence between individuals of *Hesperentomon yangi* sp. n. is 0.5% on average, between three species is 24.1% on average. The results of molecular data well support the new species identified by morphological characters.

## Supplementary Material

XML Treatment for Hesperentomon yangi
